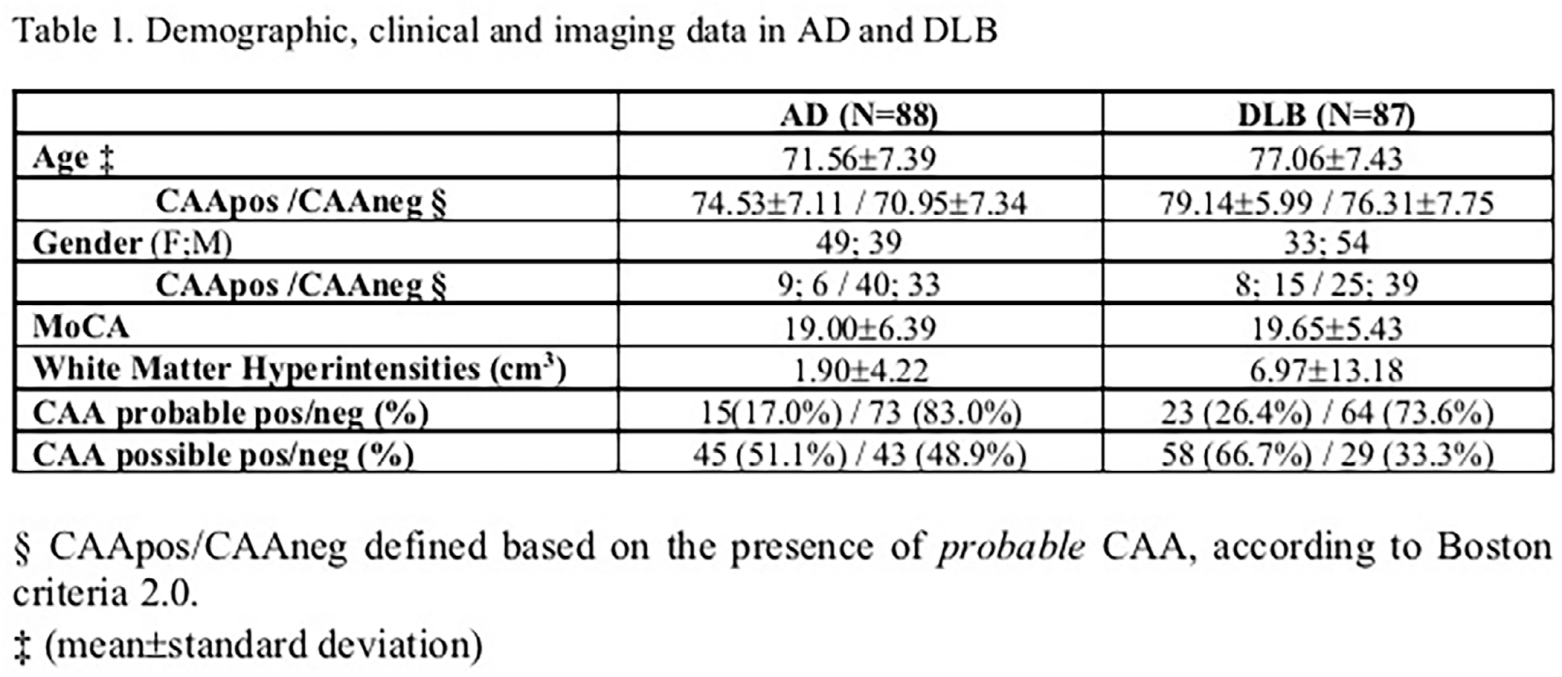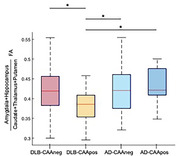# Cerebral amyloid angiopathy differently impacts gray matter microstructure in patients with Lewy bodies dementia and Alzheimer’s disease

**DOI:** 10.1002/alz.090684

**Published:** 2025-01-09

**Authors:** Giulia Bommarito, Alessandra Griffa, Caroline Hall, Yasser Aleman‐Gomez, Patric Hagmann, Gilles Allali

**Affiliations:** ^1^ Department of clinical neurosciences, Lausanne University Hospital and University of Lausanne (CHUV‐UNIL), Lausanne Switzerland; ^2^ Medical Image Processing Laboratory, Neuro‐X Institute, Ecole Polytechnique Fédérale De Lausanne (EPFL), Lausanne Switzerland; ^3^ Leenaards Memory Center, Lausanne University Hospital and University of Lausanne, Lausanne Switzerland; ^4^ Department of Radiology, Lausanne University Hospital and University of Lausanne (CHUV‐UNIL), Lausanne Switzerland

## Abstract

**Background:**

Cerebral amyloid angiopathy (CAA) has been reported in patients with dementia with Lewy bodies (DLB) and Alzheimer’s disease (AD), with a similar prevalence from pathology studies.^1,2^ CAA typically affects posterior regions, but amyloid deposits have been observed in the striatum in patients with DLB and with hereditary CAA.^1,3^ Here, we postulated that CAA‐related amyloid pathology results in a different spatial pattern of damage in DLB with respect to AD, and we tested this hypothesis in vivo, by characterizing gray matter imaging features in patients with DLB and AD with and without CAA (CAApos, CAAneg).

**Method:**

In this retrospective case‐control study, we included patients referring to the Leenaards Memory Center (Lausanne University Hospital), with a biological diagnosis of AD and a clinical diagnosis of DLB. Co‐occurrence of CAA was defined according to Boston criteria 2.0. Structural and diffusion‐weighted MRI data were processed, deriving volume, average fractional anisotropy (FA) and mean diffusivity (MD) for 13 cortical and subcortical regions.^4^ Multiple comparison correction was applied to all statistical tests.

**Result:**

Eighty‐eight AD patients AD and 87 DLB patients were included (Table 1). Among AD and DLB patients, 15 (17%) and 23 (26.4%) presented with probable CAA, respectively (p= .132). MD and FA values of putamen, caudate and thalamus were significantly higher in DLB compared to AD patients (t(173) ranging from 2.05 to 2.91, p values ranging from .027 to .033 after multiple comparison correction).

When comparing the 4 groups (AD‐CAApos, AD‐CAAneg, DLB‐CAApos, DLB‐CAAneg) subcortical microstructure significantly differed, and the ratio of limbic over basal ganglia FA values differentiated DLB‐CAApos from DLB‐CAAneg, AD‐CAAneg or AD‐CAApos (ANOVA F(3,171)= 3.44, p= .018, post‐hoc test corrected p values ranging from .021 to .041, Figure 1). No significant differences were found for posterior (occipital and parietal) regions metrics.

**Conclusion:**

As expected, patients with DLB and AD present a similar prevalence of CAA co‐pathology. However, in DLB patients, CAA associates with an increased basal ganglia over limbic anisotropy that could reflect higher iron content^5^ and is not observed in AD and DLB without CAA. These results might suggest that alpha‐synuclein and plaque amyloid depositions differently interact with CAA pathogenic pathway.